# Attributing Mind to Groups and Their Members on Two Dimensions

**DOI:** 10.3389/fpsyg.2019.00840

**Published:** 2019-04-24

**Authors:** Tetsushi Tanibe, Takaaki Hashimoto, Tobu Tomabechi, Taku Masamoto, Kaori Karasawa

**Affiliations:** ^1^Graduate School of Humanities and Sociology, The University of Tokyo, Tokyo, Japan; ^2^Japan Society for the Promotion of Science, Tokyo, Japan

**Keywords:** group mind, entitativity, mind attribution, mind perception, agency, experience, social cognition

## Abstract

Psychological research has revealed that people attribute mental states to groups such as companies, especially to those groups that are highly entitative. Moreover, attributing a mind to a group results in the decreased attribution of mind to individual group members. Recent research has demonstrated that the minds of others are perceived in two dimensions—agency and experience. The present study investigated the possibility that this two-dimensional structure exists in mind attribution to groups, and group entitativity has different patterns of relations with these dimensions. A vignette experiment revealed that highly entitative groups were attributed both agency and experience to greater degrees compared to non-entitative groups, while group entitativity reduced only the attribution of agency to the individual group members. Individual members were attributed an equivalent amount of experience regardless of group entitativity. Mind attribution to individual members showed an unpredicted third factor of other-recognition, which was positively related to group entitativity. The implications of mind attribution to moral issues were discussed.

## Introduction

### Attribution of Mind to a Group and Its Members

In our daily life, we use expressions such as “the company is *suffering* from a recession” or “the government *decided* it” as if the company and the government possess mental capacities to feel pain or make decisions. Indeed, psychological research has demonstrated that people spontaneously infer groups’ “mental” states when explaining or predicting their behaviors (O’Laughlin and Malle, 2002; Jenkins et al., 2014). However, people do not always attribute minds to groups. Rather, individuals often explain an event based on individual persons’ mental states—for example, “the company’s workers are suffering from pay cut” or “the government officials agreed on the new policy.” In these examples, the perceivers appear to focus on individual actors and pay less attention to the group as a whole. These examples suggest that people may attribute mind to a group when the group as a whole is perceived as a single entity, rather than an aggregate of individual entities that independently possess their own mental states. In other words, a group may need to be perceived as a unified entity to be perceived as possessing a mind. Regarding this point, *entitativity* will play a significant role. Entitativity is a concept proposed by Campbell (1958), which indicates the degree to which a group has real existence. Campbell (1958) argued that a group has high entitativity when its members are close and similar to each other and/or share a common fate or goal. Groups with high entitativity have been shown to be attributed more mind than non-entitative groups, as such groups are arguably likely to appear as unified agents that act on joint reasons shared by group members (O’Laughlin and Malle, 2002; Waytz and Young, 2012). Additionally, recent evidence indicates that group entitativity affects mind attribution to the group members—a phenomenon called the group-member mind trade-off (Waytz and Young, 2012). High entitativity not only facilitates the attribution of mind to a group but also reduces mind attribution to its individual members (also see Morewedge et al., 2013; Takahashi and Watanabe, 2015). The group-member mind trade-off can be plausibly understood from the standpoint of parsimony in social cognition. If individuals are able to explain a group’s behavior based on the mental state of the group, they will cease paying attention to the minds of individual members in order to maintain a parsimonious appraisal of the environment. Yzerbyt et al. (2001) have argued that stereotypes—that is, perceiving a social group as homogenous—help perceivers to understand complicated environments such that perceiving high group entitativity facilitates stereotyping. This implies that paying attention to the group as a whole and underestimating the uniqueness of individual group members enables perceivers to parsimoniously understand an event involving the group, particularly when a group is highly entitative. Although Yzerbyt et al. (2001) posit that individual persons have minds and focus on the similarities and differences between group members’ minds, it is conceivable that individual members may be perceived as mindless entities by perceivers that refrain from attending to their minds. When perceivers attribute mind to the group and understand an event through the group’s mental states, they may reduce the amount of mind they attribute to the individual group members.

### Dimensions of Mind Attribution

Given the previous discussion of mind attribution to groups and individuals, it is important to consider what types of mental capacities are attributed to groups. According to Gray et al. (2007), people perceive minds according to two dimensions: *agency*, the capacity to intend and act, and *experience*, the capacity for sensation and feelings (also see Gray and Wegner, 2012; Gray et al., 2012). This multidimensionality has not been fully considered in previous research on group mind attribution. Specifically, Waytz and Young (2012), who examined the relations between entitativity and group mind attribution, defined mind as “the capacity to make plans, have intentions, and think for itself,” which only refers to agency. On the other hand, Morewedge et al. (2013) summarized various components (beliefs, desires, consciousness, intelligence) in a single index of mind and did not distinguish between agency and experience. Although a few of recent studies distinguished between these two dimensions (Rai and Diermeier, 2015; Tang and Gray, 2018), they did not address the factor of entitativity. In sum, no research has tested the effect of group entitativity on mind attribution in terms of agency and experience. Thus, there is the unexplored possibility that entitativity differentially impacts the attribution of agency and experience to groups.

Finding that relation between mind attribution and entitativity differs for experiential versus agentic mind would change our current understanding of the group-member mind trade-off and have moral implications for groups. Literature on moral psychology argues that different dimensions of mind attribution relate to moral judgments in distinctive patterns: agency attribution leads to the attribution of moral responsibility (blaming and punishing) while experience attribution leads to the attribution of moral rights (helping and protection from harm; e.g., Gray et al., 2007, 2012). Considering that people engage in moral judgments toward groups (e.g., blaming a company for its misconduct), our study will provide insight into the important question of whether and how the multidimensional structure of mind attribution applies to decisions involving groups.

### Purpose

The present study aims to break down mind attribution into agency and experience and investigate how entitativity affects the attribution of each dimension to groups and members.

For the dimension of agency, we expect to replicate Waytz and Young (2012) findings that entitativity has opposing effects on the attribution of agency to groups and members. First, high group entitativity will facilitate attribution of agency to groups. One of the functions of mind attribution is to predict and/or control the behavior of an entity (Epley et al., 2007, 2008; Waytz et al., 2010), and agency is particularly pertinent to this function. Agency is the capacity to evoke action; therefore, the attribution of agency determines to whom an action is attributed. Perceiving high entitativity in a group contributes to the attribution of a group’s agency, as members of an entitative group share common goals and intentions, and perceiving such a group as a unified agent aids in explaining its behaviors more efficiently than perceiving it as an aggregate of independent agents.

Second, attributing agency to a group will reduce the attribution of agency to its members. When a plausible cause is present for a given event, social perceivers tend to discount the importance of other causes (Kelley, 1971); once the group is identified as an agent that has induced action and can be attributed the event, perceivers will no longer need to see individual members as agents, and will reduce the attribution of agency to them. Thus, our hypotheses regarding agency attribution are as follows:

Hypothesis 1a: Entitative groups will be attributed more agency than non-entitative groups.
Hypothesis 1b: Members of entitative groups will be attributed less agency than members of non-entitative groups.

In terms of the experience dimension, it is probable that a highly entitative group will be attributed more experience than a non-entitative group. Members of an entitative group are purported to share common interests and are likely to respond in a similar way (e.g., express pleasure, anger, etc.) to a specific event that involves that shared interests. Those members’ collective responses might lead to the perception that the group is feeling pleasure, anger, or other feelings as a consequence of the event. Although previous research has shown that experience is usually attributed less to groups than to individual persons (Knobe and Prinz, 2008; Rai and Diermeier, 2015), a recent finding suggested that experience can be attributed to groups in some situations. Tang and Gray (2018) found that experience is attributed to an organization when it is represented by its CEO, who is perceived as the human embodiment of the organization. That finding suggests that people may perceive a CEO—a human possessing agency and experience who is seen as embodying the organization—as conferring experiential mental capacity upon an organization. As such, even ordinary members might be seen as conferring experience upon the group when their mental states are perceived as embodying the group as a whole. Since members of highly entitative groups are likely to be stereotyped (Yzerbyt et al., 2001), entitativity should thus be a key factor that enables the conferring of experiential mind. In other words, individual members of highly entitative groups tend to be seen as representing the group’s underlying traits; therefore, perceiving individual members’ minds might lead to perceiving the group mind as a whole. Accordingly, we propose the following hypothesis regarding experience attribution:

Hypothesis 2: Entitative groups will be attributed more experience than non-entitative groups.

Meanwhile, regarding experience attribution to individual group members, there can be two different predictions. One possibility is that, similar to agency attribution, entitativity relates negatively to the attribution of experience to individual members. Perceivers may allocate a finite amount of mind to any entities around them, regardless of the dimension of mind. Further, since attributing experience to an organization engaged in wrongdoing makes the punishment more satisfying (Tang and Gray, 2018), perceivers might not need to seek another experiential mind when the group’s mind helps them understand a social event (e.g., punishment for misdeeds) in a meaningful way. The other possibility is that, unlike agency, the group-member trade-off will not occur in the attribution of experience and the individual members will be viewed as having the capacity for experience regardless of their group’s entitativity. This prediction is derived from the differences in the function of agency and experience. As discussed above, the group-member trade-off in agency attribution is predicted based on the function of agency that evokes action in the entity; identifying one agent is sufficient to explain or predict one action, and perceivers do not need to attribute agency to two entities—a group and its members. However, this mechanism of the trade-off might not be applicable to the attribution of experience. Experience is the capacity for feelings, and more than one entity can have the same mental state as a consequence of one event. Therefore, we do not develop a concrete hypothesis regarding the attribution of experience to individual group members; rather, we exploratorily examine the effect of entitativity.

To investigate these issues, we conducted a vignette experiment in which we manipulated the entitativity of the groups. For generalizability, we used vignettes describing two types of groups: a club in a university and a private company. We used a club because previous research that involved manipulating entitativity (Waytz and Young, 2012, Study 3) had done the same, and our first aim was to extend those findings to the experience dimension. We used a company because companies exemplify formal organizations, in which members work systematically according to specified roles and responsibilities; such features are not found to the same extent in social clubs. Further, Study 1 in Waytz and Young (2012) found that companies were attributed moderate levels of entitativity, suggesting that they are suitable for manipulating entitativity and examining its effect on mind attribution.

## Materials and Methods

### Experiment Design

We designed a three-factor experiment. First, there was a between-participant factor: group entitativity (two levels; high vs. low). Then, there were two within-participant factors: target of attribution (two levels; group vs. individual members) and the dimension of mind (two levels; agency vs. experience). The dependent variables were the degrees of mental capacity participants attributed to each target in each dimension.

Moreover, we added the difference between the vignettes (club vs. company) to the analysis as a within-participant factor to test the potential effect that the characteristics of these groups might have on mind attribution. As such, the analyses were conducted with a four-factor design.

### Participants

Previous research examining the effect of entitativity on the attribution of group mind (Waytz and Young, 2012; especially Study 3, which manipulated the entitativity of student clubs by vignettes) reported medium to large effect sizes (*d*s > 0.46).^1^ Given this effect size, we conducted a power analysis using PANGEA ver. 0.2^2^ (Westfall, 2016). We calculated the sample size required for an ANOVA with entitativity (two levels; high vs. low), target of attribution (two levels; group vs. members), and the dimension of mind (two levels; agency vs. experience) as fixed factors and the participant as a random factor nested in entitativity. The analysis showed that, to detect the interaction of entitativity × target × dimension with a medium effect size of *d* = 0.45 and a power of 0.8, at least 34 participants were needed in high- and low-entitativity conditions. However, because the attribution of experience dimension was not investigated in previous research, and thus we could not predict a particular effect size *a priori*, and because we had the additional purpose of testing potential differences between vignettes, we decided to recruit as many participants as possible, above the suggested number.

In total, 117 undergraduate or graduate students (67 females and 50 males) from universities in the Tokyo metropolitan area participated in the experiment. They volunteered to participate without compensation. Their age range was 18–32 years (*Med* = 20; *M* = 20.43; *SD* = 1.54).

### Materials

All study materials were provided in Japanese. The questionnaire included two subsets consisting of a vignette and a set of questions concerning fictitious groups. One vignette described a club in a university, and the other described a private company (a food manufacturer). These descriptions were constructed in terms of the presence of common goals and the frequency of interaction among members, serving as the experimental manipulation of group entitativity. In the club vignette, members of a highly entitative club provided advice to each other and worked together to enhance their skills. Furthermore, members often interacted at recreational events in addition to club activities. In the low-entitativity condition, on the other hand, each member recognized his/her own challenges and concentrated on enhancing their skills. Members did not partake in recreational events as frequently.

In the company vignette, the high-entitativity condition describes the company’s departments cooperating to perform their jobs. Workers reported the progress of their jobs in a meeting and they were aware of each other’s circumstances. Members often engaged in study sessions and interactions with each other. In the low-entitativity condition, the departments function independently, and workers did not report their progress in detail and were not aware of each other’s circumstances. Members only occasionally participated in study sessions and had infrequent interactions. See Supplement S1 for original and English versions of the vignettes.

### Procedure

The experiment was conducted using a paper-and-pencil questionnaire. The researchers distributed questionnaires to acquaintances who agreed to participate. Participants answered the questionnaires at their leisure and returned them to researchers at a later date.

Participants were randomly assigned to one of two conditions, high- or low-entitativity. Each participant was presented with vignettes of the same condition across both vignettes. Two subsets were presented in a counterbalanced order.

In each subset, participants read a vignette describing a group, and subsequently answered questions. The first three items concerned the manipulation check, asking participants to rate how group members were willing to belong to the group, to what extent group members shared common goals, and to what extent group members interacted with each other, using a seven-point scale (from 1 = “*disagree*” to 7 = “*agree*”). Next, participants rated the extent to which the group possessed 22 mental capacities^3^, using a five-point scale (from 1 = “*disagree*” to 5 = “*agree*”). Finally, they rated the extent that individual group members possess those mental capacities.

### Ethics Statement

Recruitment and study procedures conformed to the requirements of the Declaration of Helsinki. The study was approved by the Research Ethics Committee from the Department of Social Psychology, The University of Tokyo.

All participants were informed that their participation was fully based on their free will and that the data would be processed anonymously. We provided this information on the first page of the questionnaire and asked participants to proceed to the subsequent survey only if they agreed with the instructions. Therefore, their participation was taken as agreement with the instruction and as assent to participate.

## Results

The data analyses were conducted using R ver. 3.5.0 as well as HAD ver. 16.056, a free software program for statistical analysis in psychology (Shimizu, 2016).

### Factor Structure of Mind Attribution

We conducted exploratory factor analyses (EFAs) with the maximum likelihood method and promax rotation on the mind attribution data. We conducted the same analyses separately on the ratings of four targets (i.e., the club, the club members, the company, and the company members). The scree plots indicated that two-factor structures were suitable for mind attribution to groups (the club and the company), while three-factor structures were suitable for mind attribution to individuals (club members and company members). We followed these indications and extracted either two or three factors accordingly. Then, we omitted three items that did not load on the predicted factors (see Supplement S3 and Supplement Table S1 for details). All four analyses found that the two factors of *agency* and *experience* were consistent with the factor structure proposed by Gray et al. (2007). Additionally, the factor analyses of the ratings of mind attribution to individuals found a third factor which we labeled *other-recognition*. Items consisting of the other-recognition factor all loaded on the agency factor in group mind; this suggested that other-recognition is a subcategory of agentic mental capacity, which is also consistent with Gray et al. (2007). The third factor was one that we did not expect prior to data collection, but it seems worthy of exploratory investigation. We speculate that capacity of other-recognition is different from other agentic aspects of mind (e.g., intention) in that the capacity becomes salient specifically when an entity interacts with another entity within one’s network. Such inter-relational nature of the capacity may have caused the perception of it being a unique aspect reflecting the entity’s mental capacity especially when the attribution target was members within a group. In Section “Discussion,” we explain in detail why these items may have comprised a new factor distinct from agency.

Thus, we composed mind attribution indices to be used for hypothesis testing based on the factor structures found by EFAs. Although the results of EFAs indicated different numbers of factors for the group mind and individual member mind, they did not contradict one another given that the other-recognition factor is a subcategory of agency. Moreover, we conducted confirmatory factor analyses to examine the goodness of fit of two- and three-factor structures and found consistently that the three-factor structure fitted the data better than the two-factor structure, suggesting reasonability in comparing mind attribution to groups and to individual members based on the same three-factor structure (see Supplement S3 and Supplement Tables S2, S3). Therefore, we composed the three indices of agency, other-recognition, and experience for each of the four rating targets. Table 1 lists the items comprising each index and their internal consistency (Cronbach’s α). Internal consistency was high for all indices, and therefore the ratings were averaged to comprise each index. Mean scores and standard deviations of the composite indices are also reported in Table 1.

**Table 1:** Items for composing mind attribution indices.

Dimension of mind	Items	Club-group		Club-members		Company-group		Company-members	
		α	Mean (SD)	α	Mean (SD)	α	Mean (SD)	α	Mean (SD)
Agency	Reflect, memory, predict, planning, self-control, morality, thought, decision-making	0.899	3.54 (0.67) 2.40 (0.60)	0.861	3.36 (0.63) 3.88 (0.50)	0.906	3.85 (0.54) 2.62 (0.75)	0.802	3.43 (0.61) 3.64 (0.54)
Other-recognition	Emotion-recognition, intention-recognition, communicating	0.863	3.59 (0.75) 2.21 (0.65)	0.807	3.82 (0.73) 3.07 (0.71)	0.883	3.76 (0.80) 2.19 (0.76)	0.838	3.76 (0.71) 2.88 (0.80)
Experience	Pain, embarrassment, joy, fear, hesitation, upset, anger, sad	0.901	3.24 (0.62) 2.58 (0.91)	0.887	3.60 (0.73) 3.74 (0.72)	0.861	3.18 (0.54) 2.65 (0.77)	0.897	3.59 (0.63) 3.58 (0.69)

*Mean scores and standard deviations (SD) were calculated for the high-entitativity condition (top rows) and the low-entitativity condition (bottom rows), respectively.*

### Manipulation Check

Manipulation checks were conducted before the main analyses. Responses to the three manipulation check items (α = 0.77 for the club vignette and 0.78 for the company vignette) were averaged to form an index of the perception of entitativity. Welch’s *t*-test revealed that the manipulation of entitativity was effective in both vignettes. In the club vignettes, the club with high entitativity was rated as more entitative (*M* = 5.73, *SD* = 0.91) than the club with low entitativity [*M* = 3.24, *SD* = 0.86; *t*(115.00) = 15.03, *p* < 0.001]. Likewise, in the company vignette, the company with high entitativity was rated as more entitative (*M* = 5.49, *SD* = 0.82) than the company with low entitativity [*M* = 3.09, *SD* = 1.03; *t*(107.06) = 13.77, *p* < 0.001].

### Effect of Entitativity on Mind Attribution to Groups and Members

First, we conducted a four-way analysis of variance (ANOVA) to test the effect of entitativity on mind attribution to groups and individual members. The independent variables were entitativity (high vs. low) as a between-participant factor and target of attribution (group vs. member), dimension of mind (agency vs. other-recognition vs. experience), and vignette (club vs. company) as within-participant factors. Although we did not predict any effect of the vignette since we were testing the same hypotheses using two types of vignettes to affirm the generalizability of the results, we used it as a within-participant factor to test the possibility that entitativity had different patterns of effects on different types of groups.

Supplement Table S4 reports the overall ANOVA results. Regarding our hypotheses, there was a significant three-way interaction of entitativity × dimension of mind × target of attribution [*F*(2,224) = 30.31, *p* < 0.001, $\eta_{p}^{2}$ = 0.213]. This interaction effect was not moderated by the difference between vignettes—that is, the four-way interaction was not significant [*F*(2,224) = 1.85, *p* = 0.159, $\eta_{p}^{2}$ = 0.016]. Other interaction effects that included both the vignette and entitativity were all non-significant (*p*s > 0.129), indicating that the effect of entitativity on mind attribution had the same pattern in both vignettes. For ease of comprehension, we conducted *post hoc* simple interaction analyses separated by vignette; the results are reported below.

#### Club Vignette

In the *post hoc* analysis for the club vignette, the three-way interaction effect of entitativity × target × dimension was significant [*F*(2,224) = 11.42, *p* < 0.001, $\eta_{p}^{2}$ = 0.093]. Thus, we subsequently conducted tests for the simple effects of entitativity and the target of attribution on the attribution of each dimension of mind.

Regarding the attribution of agentic mind, the interaction effect of entitativity × target was significant [*F*(1,112) = 191.34, *p* < 0.001, $\eta_{p}^{2}$ = 0.631]. Subsequent tests for the simple main effects revealed that entitativity had significant effects on both group agency and member agency attribution, but in the opposite direction. Attribution of agency to the club with high entitativity was higher than to the club with low entitativity [*F*(1,112) = 115.15, *p* < 0.001, $\eta_{p}^{2}$ = 0.507]. Meanwhile, agency attribution to individual club members was lower when entitativity was high than when it was low [*F*(1,112) = 24.28, *p* < 0.001, $\eta_{p}^{2}$ = 0.178]. Focusing on the simple main effect of the target of attribution, the club with high entitativity was attributed more agency than its members [*F*(1,56) = 10.58, *p* = 0.002, $\eta_{p}^{2}$ = 0.159] whereas the club with low entitativity was attributed less agency than its members [*F*(1,56) = 213.31, *p* < 0.001, $\eta_{p}^{2}$ = 0.792].

Next we considered the effects of entitativity and the attribution target on the attribution of other-recognition. The interaction effect of entitativity × target was again significant [*F*(1,112) = 22.87, *p* < 0.001, $\eta_{p}^{2}$ = 0.170]. Entitativity was positively related to both the club’s and individual members’ other-recognition attribution, though the effect size was larger in the attribution to the club. The attribution of other-recognition to the club with high entitativity was higher than to the club with low entitativity [*F*(1,112) = 129.89, *p* < 0.001, $\eta_{p}^{2}$ = 0.537]. Similarly, the attribution of other-recognition to individual club members was higher when entitativity was high than when it was low [*F*(1,112) = 37.15, *p* < 0.001, $\eta_{p}^{2}$ = 0.249]. More other-recognition capacity was attributed to individual members than to the club, regardless of entitativity [high entitativity: *F*(1,56) = 8.02, *p* = 0.006, $\eta_{p}^{2}$ = 0.125; low entitativity: *F*(1,56) = 71.75, *p* < 0.001, $\eta_{p}^{2}$ = 0.562].

Lastly, we analyzed the effects of entitativity and the attribution target on experiential mind attribution. The interaction effect of entitativity × target was significant [*F*(1,112) = 21.93, *p* < 0.001, $\eta_{p}^{2}$ = 0.164]. The tests for simple main effects revealed that entitativity affected only the attribution of experience to the club. The club with high entitativity was attributed more experience than the club with low entitativity [*F*(1,112) = 20.78, *p* < 0.001, $\eta_{p}^{2}$ = 0.157] whereas club members were attributed equal amounts of experience regardless of entitativity [*F*(1,112) = 1.45, *p* = 0.231, $\eta_{p}^{2}$ = 0.013]. Individual members were attributed more experiential mind than the club regardless of entitativity [high entitativity: *F*(1,56) = 13.94, *p* < 0.001, $\eta_{p}^{2}$ = 0.199; low entitativity: *F*(1,56) = 59.01, *p* < 0.001, $\eta_{p}^{2}$ = 0.513].

**FIGURE 1:**
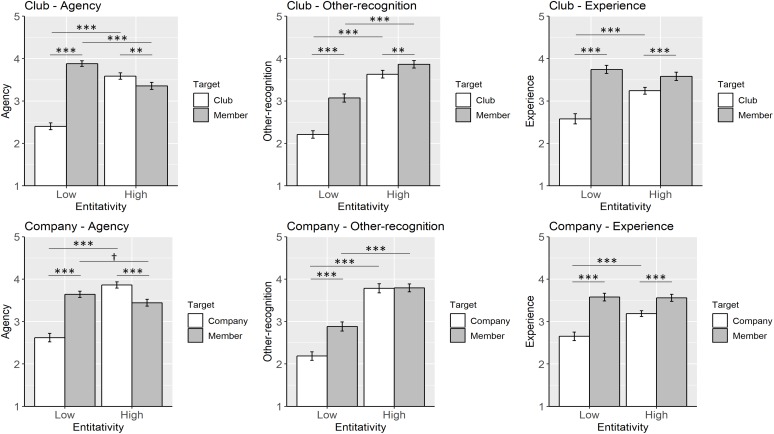
Mind attribution to groups and individual members. *Note*. Error bars indicate standard errors. Statistical significances are marked as ^∗∗∗^*p* < 0.001, ^∗∗^*p* < 0.01, and †*p* < 0.10.

The top row in Figure 1 summarizes the analysis results for the club vignette.

#### Company Vignette

Similar to the club case, the three-way interaction effect of entitativity × target × dimension was significant [*F*(2,224) = 9.03, *p* < 0.001, $\eta_{p}^{2}$ = 0.075]. Thus, we subsequently conducted tests for the simple effects of entitativity and the target of attribution on the attribution of each dimension of mind.

Regarding the attribution of agentic mind, the interaction effect of entitativity × target was significant [*F*(1,112) = 115.17, *p* < 0.001, $\eta_{p}^{2}$ = 0.507]. The tests for simple main effects revealed that, similar to the club vignette, participants attributed more agency to the company with high entitativity than the one with low entitativity [*F*(1,112) = 101.67, *p* < 0.001, $\eta_{p}^{2}$ = 0.476]. The effect of entitativity on individual members’ agency attribution was in the opposite direction, though the statistical significance was marginal, indicating that members of the highly entitative company were attributed less agency than members of the non-entitative company [*F*(1,112) = 3.41, *p* = 0.068, $\eta_{p}^{2}$ = 0.030]. Regarding the effects of the attribution target, participants attributed more agency to the company than to its members when entitativity was high [*F*(1,56) = 34.39, *p* < 0.001, $\eta_{p}^{2}$ = 0.380] while less agency was attributed to the company than to its members when entitativity was low [*F*(1,56) = 80.81, *p* < 0.001, $\eta_{p}^{2}$ = 0.591].

Next, regarding the attribution of other-recognition, the interaction effect of entitativity × target was significant [*F*(1,112) = 20.00, *p* < 0.001, $\eta_{p}^{2}$ = 0.152]. The tests for simple main effects revealed that participants attributed more other-recognition capacity to both the company and its members when entitativity was high than when it was low [company: *F*(1,112) = 118.29, *p* < 0.001, $\eta_{p}^{2}$ = 0.514; members: *F*(1,112) = 41.62, *p* < 0.001, $\eta_{p}^{2}$ = 0.271]. Regarding the simple main effects of the attribution target, more other-recognition capacity was attributed to individual members of the company with low entitativity than to the company [*F*(1,56) = 32.16, *p* < 0.001, $\eta_{p}^{2}$ = 0.365]. Meanwhile, as the only difference from the club vignette, the company and its members were attributed an equal amount of other-recognition capacity when entitativity was high [*F*(1,56) = 0.02, *p* = 0.899, $\eta_{p}^{2}$ < 0.001].

Lastly, regarding experiential mind, the interaction effect of entitativity × target was again significant [*F*(1,112) = 12.37, *p* = 0.001, $\eta_{p}^{2}$ = 0.100]. The tests for simple main effects found the same pattern of effects as the club vignette. Participants attributed more experience to the company with high entitativity than the company with low entitativity [*F*(1,112) = 19.00, *p* < 0.001, $\eta_{p}^{2}$ = 0.145], while individual company members were attributed an equal amount of experience regardless of entitativity [*F*(1,112) = 0.02, *p* = 0.902, $\eta_{p}^{2}$ < 0.001]. In addition, individual members were attributed more experience than the company regardless of entitativity [high entitativity: *F*(1,56) = 17.06, *p* < 0.001, $\eta_{p}^{2}$ = 0.234; low entitativity: *F*(1,56) = 52.70, *p* < 0.001, $\eta_{p}^{2}$ = 0.485].

The bottom row in Figure 1 summarizes the analysis results for the company vignette.

#### Differences Between Vignettes

According to the results of the four-way ANOVA reported above, the three-way interaction between vignette, dimension of mind, and target of attribution was marginally significant [*F*(2,224) = 2.56, *p* = 0.079, $\eta_{p}^{2}$ = 0.022]. This suggests that the patterns of mind attribution were different between the vignettes, though they are not directly related to our hypotheses. To investigate the differences between vignettes in more detail, we conducted *post hoc* analyses separated by dimension of mind. Note that we do not focus on the effects of entitativity here since no interaction effects involving entitativity and vignette were significant, indicating that entitativity had the same pattern of effects in both vignettes.

We first conducted the *post hoc* analysis regarding the attribution of agentic mind. The interaction of vignette × target was significant [*F*(1,112) = 17.07, *p* < 0.001, $\eta_{p}^{2}$ = 0.132]. The tests for simple main effects revealed that participants attributed more agency to the company than the club [*F*(1,112) = 15.26, *p* < 0.001, $\eta_{p}^{2}$ = 0.120], though the difference was small, whereas they attributed equal amounts of agency to company members and club members [*F*(1,112) = 2.05, *p* = 0.155, $\eta_{p}^{2}$ = 0.018]. Individual members were attributed more agency than the group in both vignettes [club vignette: *F*(1,112) = 102.08, *p* < 0.001, $\eta_{p}^{2}$ = 0.477; company vignette: *F*(1,112) = 19.97, *p* < 0.001, $\eta_{p}^{2}$ = 0.151].

Second, *post hoc* analysis was conducted regarding the attribution of other-recognition. The interaction of vignette × target was again significant [*F*(1,112) = 4.18, *p* = 0.043, $\eta_{p}^{2}$ = 0.036]. Club members were attributed slightly more other-recognition capacity than company members [*F*(1,112) = 3.25, *p* = 0.074, $\eta_{p}^{2}$ = 0.028]. The club and the company as groups were attributed the same degree of other-recognition [*F*(1,112) = 0.78, *p* = 0.378, $\eta_{p}^{2}$ = 0.007]. Individual members were attributed more other-recognition capacity than the group in both vignettes [club vignette: *F*(1,112) = 69.85, *p* < 0.001, $\eta_{p}^{2}$ = 0.384; company vignette: *F*(1,112) = 21.39, *p* < 0.001, $\eta_{p}^{2}$ = 0.160].

Third, the attribution of experiential mind was analyzed. The main effect of the target of attribution was significant [*F*(1,112) = 209.63, *p* < 0.001, $\eta_{p}^{2}$ = 0.652], indicating that individual members were attributed more experience than the group in both vignettes. The main effect of vignette [*F*(1,112) = 0.34, *p* = 0.563, $\eta_{p}^{2}$ = 0.003] and the interaction of vignette × target of attribution [*F*(1,112) = 1.42, *p* = 0.236, $\eta_{p}^{2}$ = 0.013] were not significant.

## Discussion

### Dimensions of Mind Attributed to Groups and Individual Members

In both cases, the results of the factor analyses demonstrated that perceivers attribute minds to groups in terms of agency and experience. This suggests that we can place a group on the two-dimensional map proposed by Gray et al. (2007) and compare it with other entities on the map. Although a few existing studies measured group mind attribution based on the agency-experience distinction (Rai and Diermeier, 2015; Tang and Gray, 2018), they did not directly examine the factor structure. The present study showed that the two-dimensional structure of agency and experience applied to group mind attribution and gave support to the validity of this distinction.

In addition, a third factor, other-recognition, was found in the attribution of individual group members’ minds. The mental capacity for other-recognition is different from other components of agentic mind (e.g., self-control) in that it is needed only when an individual interacts with other entities; this capacity should be particularly important when an individual is perceived as a member of the group because members would be expected to exchange information with other members and behave in accordance with the group’s shared goals. This capacity would be less salient when individuals behave independently and outside the group context; in such cases, the capacity might be indistinguishable from other agentic capacities related to autonomous action (e.g., thinking, planning). On the other hand, in the present study wherein perceivers evaluate the mental capacities of those who act within a group, the aspect of other-recognition may have been more salient, fostering a perception distinct from other items of agency.

The other-recognition factor was not found in previous studies (Rai and Diermeier, 2015; Tang and Gray, 2018) perhaps because they measured mind attribution by a small set of items that covered agency and experience dimensions based on previous literature and did not newly examine the factor structure specifically to the group and group-member mind attribution. Having said that, our finding is only based on a single study and could be specific to the scenarios or procedures used in the study (as also discussed in section “Entitativity and Attribution of Other-Recognition”); thus, more data from other studies are needed to examine the robustness of the distinction between other-recognition and the other dimensions of mind.

### Entitativity and Attribution of Agency and Experience

The results of ANOVAs supported Hypotheses 1a and 2 by showing that entitative groups were attributed both agency and experience to a greater extent than non-entitative groups. Previous research has shown the effect of entitativity on mind attribution to groups in terms of agency (Waytz and Young, 2012), and the present study extended this finding to the dimension of experience. As discussed in Section “Introduction,” high entitativity can lead to perceiving individual members in a stereotyped way, implying that they embody the entire group’s traits. This perception could enable attributing experiential mind conferred by members to the group as a whole, similar to the effect of the CEO representing the organization (Tang and Gray, 2018).

We should note, however, that the rating for experiential mind attribution was moderate (mean score: about three on a five-point scale, even when entitativity was high). In line with previous findings that groups are attributed low levels of experience (Knobe and Prinz, 2008; Rai and Diermeier, 2015), participants in the present study did not fundamentally attribute experience to groups. Further, although attribution increased when entitativity was high, it was not above the midpoint on the scale, indicating that it was neither high nor low.

Next, regarding the attribution of mind to individual group members, group entitativity showed different patterns of effects on the dimensions of agency and experience. That is, the group-member mind trade-off occurred only in the agency dimension. The comparison of the attribution of agency to groups and members supported Hypotheses 1a and 1b by showing a group-member trade-off; people attributed more agency to entitative groups and less agency to their members. This result was consistent with Waytz and Young’s (2012) findings. However, there was no such trade-off in the attribution of experience. While entitative groups were attributed more experience than non-entitative groups, entitativity did not affect the attribution of experience to their members. As mentioned in Section “Introduction,” the discrepancy between agency and experience is based on the different functions of each dimension of mind; agency is the mental capacity to evoke action and one agentic entity is sufficient to cause one action; whereas experience is the mental capacity for feelings and more than one entity can be viewed as possessing it in a single event.

The group-member trade-off observed in agency attribution can be plausibly interpreted if social perceivers have a need to understand the environment surrounding them parsimoniously and they attribute mind to entities in the environment as a means to understand the entities’ behaviors. This is a mechanism that Waytz and Young (2012) called the “economy of mind,” wherein perceivers are capable of attributing a finite amount of mind to entities. Agency is the mental capacity to evoke action, and therefore attributing it to entities should provide perceivers with a means to understand their actions on the basis of their inner states such as goals and intentions. For example, when a company engages in misconduct (e.g., a food manufacturer sells products containing harmful chemicals), people want to know who was responsible for it (a worker in the factory, a manager, the CEO, or the company as a whole). Determining who was responsible helps people understand the event in a meaningful way and how to respond to it (whom to blame, whether they should buy the company’s products, and so on). When an action is performed by a group of individuals in a highly unified manner—in other words, when a group give perceivers highly entitative impression—it would be more parsimonious for perceivers to see the action as a “group’s action” than to see it as a sum of actions by individual members; therefore, the group as a whole will be a prior target of agency attribution. Then, understanding the action as evoked by a group’s mental states will reduce the amount of agency attributed to individual members; each member’s action can be interpreted based on the group’s mental state and they will be perceived as relatively less agentic, delegating their agency to the group to some degree.

On the other hand, the group-member trade-off was not observed in the attribution of experience. Experience is the mental capacity for sensation and feelings and more likely to be attributed to an entity that receive results of any event rather than entities that evoked it (Gray and Wegner, 2009). For example, economic recession will cause suffering for a company and its workers. Inferences about the inner states of the involved entities (e.g., suffering) will inform perceivers about whether the event is positive or negative. When perceivers see an entitative group, with members that are supposed to share a common interest and have similar mental states as a consequence of an event, attributing experience to the group as a whole will enable perceivers to understand the situation involving that group in a simple way. However, our results indicate that attributing experience to the group did not mean reducing the attribution of it to individual group members. The “economy of mind” mechanism would not apply to the experience dimension probably because of the difference of perceivers’ motivation between agency attribution and experience attribution. Perceivers pay attention to entities’ experiential capacity not to identify the source of actions but to know consequences of those actions. Whereas one action is attributed to one agent, the same action can affect more than one entity simultaneously and there is no need to allocate a finite amount of experiential capacity among entities.

### Entitativity and Attribution of Other-Recognition

In the present study, we found an unpredicted factor of other-recognition. The ANOVA results showed that highly entitative groups were attributed other-recognition capacity to a greater degree than non-entitative groups. This is the same pattern of effect as the agency and experience dimensions. Because other-recognition and agency comprised one factor in the factor structure of group mind attribution (see Supplement S3), it is no surprise that the attribution of other-recognition to groups showed the same pattern as agency. A highly entitative group’s actions involving communicating with entities outside the group will be seen as evoked by the group’s other-recognition capacity in that perceivers can understand those actions parsimoniously.

Moreover, the attribution of other-recognition capacity to individual group members was also higher when entitativity was high than when it was low. This is apparently puzzling given that other-recognition should be a subcategory of agency, the dimension in which the group-member trade-off was observed. However, communication among members is a necessary element to generate or maintain entitativity. This role of communication creates a distinction between other-recognition and other agentic mental capacities since the latter (e.g., self-control, thought, and memory among individual members) do not contribute to enhancing the group’s entitativity. Therefore, high entitativity implies that members of that group possess high other-recognition capacity.

We must acknowledge, however, that these results could be attributable to our manipulations. Our manipulation of entitativity included descriptions of interaction frequency, which could imply that the members of the highly entitative groups were good at communicating while the members of the non-entitative groups had difficulty communicating because of a lack of other-recognition capacity. More work is needed to examine whether other aspects of entitativity (e.g., similarities and common fates among members) have positive relations with the attribution of other-recognition capacity to individual members.

### Mind Attribution to Groups and Moral Issues

Here, we discuss the potential moral consequences of mind attribution to groups. Previous research has shown that mind attribution is related to moral judgments, and therefore, the attribution of group mind may affect people’s attitudes toward groups. For example, the boycott of companies engaged in wrongdoing will be understood as punishment by consumers. Since agency attribution is related to the attribution of moral responsibility (Gray et al., 2007, 2012), a company’s misconduct may elicit stronger moral outrage from consumers as they attribute more agency to the company, thus leading to the motivation for punishment. This punishment will be more satisfying for consumers who also attribute experience to the company since experience attribution implies that the boycott will more effectively inflict suffering on the company (Rai et al., 2017; Tang and Gray, 2018). Therefore, given our finding that highly entitative groups are attributed both agency and experience to a high degree, the boycott as punishment will likely occur when the company engaged in wrongdoing is seen as an entitative organization.

In addition to aggressive actions such as boycotts, we can also predict the positive consequences of mind attribution. Consider charities for refugees or poor people, for instance. Findings regarding the identifiable victim effect (Small and Loewenstein, 2003) suggest that people are less motivated to help when victims are recognized as a group than as individuals. However, perceiving them as a highly entitative group may elicit motives to help since attributing experience positively relates to attributing moral rights (Gray et al., 2007, 2012). In addition, the motivation to help entitative groups is free of the side effects such as a weakened concern for individual victims. This is because, in the present study, the attribution of experience to individual group members remained at a high level when entitativity was high.

Since the present study only investigated the antecedents of mind attribution and did not deal with its consequences, these predictions are only speculation. However, such consequences should have a social impact and be worthy of further investigation.

### Limitations and Future Research

The first limitation of this study is that our participants rated the mental capacity of only two groups in the fictitious vignettes. Future research should investigate mind attribution to real groups and integrate findings of real-world studies and experimental studies into a more generalizable conclusion. Further, in the real world, there are many different types of clubs and companies, as well as other groups such as families, local communities, and even nations. Although this study examined the perceptions of two different types of groups to enhance generalizability, it was certainly not possible to cover all types of groups in a single study. We should examine whether this study’s findings can apply to other types of groups by focusing on the various aspects that characterize each of them.

Second, entitativity should be manipulated in other aspects to examine the robustness of the effect on mind attribution. In the present study, entitativity was manipulated in terms of the presence of common goals and the frequency of interaction among members. Although these elements relate to entitativity, it is likely that words such as *goal* and *interact* implied the existence of an agentic mind. It should be added, however, that the manipulation of entitativity had an effect on the attribution of experience, which was not explicitly described in the manipulation. Nevertheless, future work should manipulate entitativity in other aspects that appear to have no relation to mental capacities (e.g., physical proximity or similarities between members).

Third, the measurement items could have been understood by the participants in ways that departed from the intended meaning. This study was conducted in Japan using translated questionnaire items that were originally developed in English and used mainly for Western participants. Therefore, there could have been some unintended effects related to linguistic or cultural background. For example, some items were excluded from analysis because they did not load on the factor proposed in previous research (see Supplement S3). This could be because the translated items did not convey the same meanings as the original ones. To our knowledge, a standardized Japanese scale for mind attribution has not yet been developed; developing such a scale is thus an important issue. That said, recent findings suggest that the multidimensional structure of mind attribution found in Western samples is consistently found in Japan, although a few differences are found between studies (Takahashi et al., 2016; Kamide et al., 2017; Tanibe et al., 2017). Therefore, despite some potential cultural differences, we can say that the concept and measurement of mind attribution have a certain degree of universality.

One noteworthy question regarding cultural differences is whether the other-recognition dimension of mind is also found in cultures other than Japan. Eastern cultures, including Japan, tend to be characterized by an interdependent construal of the self—that is, the self is understood as part of social relationships or contexts (Markus and Kitayama, 1991). This view of the self is contrasts with the independent construal of the self that is prevalent in Western cultures and emphasizes individual uniqueness. The participants in the present study, who were likely familiar with the interdependent construal of the self, might have seen other-recognition capacity as an important skill for behaving properly in relations with others as well as an element of mind that differs from agency, which does not imply social relations. Future work should examine whether the mental capacity of other-recognition is also perceived as distinct from agency in the perceptions of Western people.

## Ethics Statement

Recruitment and study procedures conformed to the requirements of the Declaration of Helsinki. The study was approved by the Research Ethics Committee from the Department of Social Psychology, The University of Tokyo.

All participants were informed that their participation was fully based on their free will and that the data would be processed anonymously. We provided this information on the first page of the questionnaire and asked participants to proceed to the subsequent survey only if they agreed the instruction. Therefore, their participation was taken as agreement with the instruction and as assent to participate.

## Author Contributions

TTa, TH, TTo, TM, and KK conceived and designed the study. TTa, TTo, and TM performed the experiments. TTa analyzed the data and wrote the original draft of the manuscript. TH, TTo, TM, and KK reviewed the draft. KK supervised the research.

## Conflict of Interest Statement

The authors declare that the research was conducted in the absence of any commercial or financial relationships that could be construed as a potential conflict of interest.

## Footnotes

1 Since Waytz and Young (2012) did not report effect sizes, we calculated them from the *t*-values and degrees of freedom they reported.

2 http://jakewestfall.org/pangea/

3 The selection of items was initially based on previous research that examined mind attribution (Gray et al., 2007; Waytz and Young, 2012). Then, we omitted some items that seemed unnatural to attribute to groups (e.g., hunger) and added items that can relate to mental capacity suggested by undergraduate research assistants majoring social psychology. We asked those assistants to provide ideas to cover the broadest concept of “mind.” All items appear in Supplement S2.
