# Supplementary material for: Attributing Mind to Groups and Their Members on Two Dimensions
Source: Front Psychol. 2019 Apr 24;10:840. doi: 10.3389/fpsyg.2019.00840 (PMC6491843; doi:10.3389/fpsyg.2019.00840)
Supplement: Supplementary file 1 [file Data_Sheet_1.pdf]

doi: 10.3389/fpsyg.2019.00840

Supplementary Materials

Supplement 1: Vignettes presented in the experiment

1a. Club vignette: High entitativity

ある大学のサークルは、大学の体育館や近所の小学校の体育館を主な活動場所にしており、練習は月・水・金曜日の週 3 回行っています。活動時間は 19 時から 21 時までの 2 時間程度で、授業後に練習をしており、お互いにアドバイスし技術を高め合っています。全員が集まるミーティングをたびたび開き、お互いの課題を確認することで、メンバー全員が出場する次の大会で結果を残せるように努力しています。普段の練習以外でもメンバー同士の交流が盛んで、月に 1 回程度は企画されるイベントにはメンバーのほとんどが参加しています。

A student club at a university meets up three times a week—on Mondays, Wednesdays, and Fridays—for practice at the university's gymnasium or the gymnasium of a local elementary school. Practice sessions are held for about two hours after school, from 19:00 to 21:00, where the members give advice to each other to improve their skills. They often hold meetings where all members participate to share each other's personal goals. The members are putting forth effort to succeed at the next tournament, in which they will all participate. The members interact socially outside of practice, and almost all of them show up for recreational events held about once a month.

1b. Club vignette: Low entitativity

ある大学のサークルは、大学の体育館や近所の小学校の体育館を主な活動場所にしており、練習は月・水・金曜日の週 3 回行っています。活動時間は 19 時から 21 時までの 2 時間程度で、授業後に練習をしており、一人一人が自らの技術の向上に努め自分の課題に集中しています。全員が集まるミーティングはあまり行っておらず、各々が自身の課題に集中して、次の大会で結果を残せるように努力しています。普段の練習以外ではメンバー同士の交流はあまりなく、年に数回企画されるイベントもメンバーの一部が参加しているだけです。

A student club at a university meets up three times a week—on Mondays, Wednesdays, and Fridays—for practice at the university's gymnasium or the gymnasium of a local elementary school. Practice sessions are held for about two hours after school, from 19:00 to 21:00, where the members focus on their own tasks to improve their individual skills. They rarely hold meetings where all members participate. The members are individually putting forth effort to achieve their personal goals and to succeed at the next tournament. The members don't have a lot of social interaction outside of practice, and only a part of the team shows up for recreational events held several times a year.

2a. Company vignette: High entitativity

「まん丸フーズ」は 1983 年創業の食品メーカーで、冷凍食品を幅広く製造・販売しています。首都圏のスーパーを中心に商品を卸しており、手ごろな価格設定でありながら、質の高い商品が多く、主婦層を中心に高い評価を受けています。
社内はいくつかの部門に分かれており、異なる部門がお互いに連携しながら仕事をしています。社員による勉強会も頻繁に開かれており、社員同士の交流が盛んです。会議では社員個々人が仕事内容を詳細に報告し、他の社員がどのような仕事をしているのか、互いによく把握しています。

Man-maru Foods is a food manufacturer founded in 1983 that produces and sells a variety of frozen meals. Its products are mainly sold in grocery stores in the Tokyo metropolitan area. Its products are highly recognized by consumers for their reasonable pricing and good quality.
The company has several departments, all of which perform tasks in cooperation with one another. Employees often hold study meetings, and they interact socially a great deal. In department meetings, each member reports on their circumstances in detail, so people have a good understanding of each other's tasks.

(*Note*: "*Man-maru*" refers to the geometric figure of perfect circle. We used this word as the name of the company so that it would not create any positive or negative impressions for participants.)

doi: 10.3389/fpsyg.2019.00840

2b. Company vignette: Low entitativity

「まん丸フーズ」は 1983 年創業の食品メーカーで、冷凍食品を幅広く製造・販売しています。首都圏のスーパーを中心に商品を卸しており、手ごろな価格設定でありながら、質の高い商品が多く、主婦層を中心に高い評価を受けています。
社内はいくつかの部門に分かれており、それぞれが独立して仕事をしています。たまに一部の社員による勉強会が開かれていますが、社員同士の交流はあまり盛んではありません。会議では社員個々人の仕事内容を詳細に報告することはなく、他の社員がどのような仕事をしているのか、互いにあまり把握していません。

Man-maru Foods is a food manufacturer founded in 1983 that produces and sells a variety of frozen meals. Its products are mainly sold in grocery stores in the Tokyo metropolitan area. Its products are highly recognized by consumers for their reasonable pricing and good quality.
The company has several departments, all of which perform their tasks independently. Although some employees hold occasional study meetings, they do not interact socially a great deal. In department meetings, the members do not report on their circumstances in detail, so people do not really have a good understanding of each other's tasks.

Supplement 2: Questionnaire items for mind attribution

1. (reflect) 集団（個人）として、過去の行いを反省できる
   As a group, it is capable of reflecting on its past actions.
   As an individual, one is capable of reflecting on one's past actions.

2. (memory) 集団（個人）として、情報を記憶しておくことができる
   As a group, it is capable of remembering information.
   As an individual, one is capable of remembering information.

3. (predict) 集団（個人）として、将来を予測することができる
   As a group, it is capable of making predictions about the future.
   As an individual, one is capable of making predictions about the future.

4. (planning) 集団（個人）として、計画的に行動することができる
   As a group, it is capable of acting according to plans.
   As an individual, one is capable of acting according to plans.

5. (self-control) 集団（個人）として、状況に沿って行動を調節できる
   As a group, it is capable of controlling its actions depending on circumstances.
   As an individual, one is capable of controlling one's actions depending on circumstances.

6. (emotion-recognition) 集団（個人）として、他者が感じていることを理解できる
   As a group, it is capable of understanding others' feelings.
   As an individual, one is capable of understanding others' feelings.

7. (intention-recognition) 集団（個人）として、他者の意図を理解できる
   As a group, it is capable of understanding others' intentions.
   As an individual, one is capable of understanding others' intentions.

8. (morality) 集団（個人）として、善悪を区別し、正しく振舞おうとできる
   As a group, it is capable of telling right from wrong and trying to do the right thing.
   As an individual, one is capable of telling right from wrong and trying to do the right thing.

9. (thought) 集団（個人）として、ものごとを考えることができる
   As a group, it is capable of thinking.
   As an individual, one is capable of thinking.

10. (decision-making) 集団（個人）として、意思決定をすることができる
    As a group, it is capable of making decisions.
    As an individual, one is capable of making decisions.

11. (communicating) 集団（個人）として、コミュニケーションをすることができる
    As a group, it is capable of communicating.
    As an individual, one is capable of communicating.

12. (pain) 集団（個人）として、苦しみを経験しうる
    As a group, it is capable of experiencing pain.
    As an individual, one is capable of experiencing pain.

13. (embarrassment) 集団（個人）として、恥ずかしさを経験しうる
    As a group, it is capable of experiencing embarrassment.
    As an individual, one is capable of experiencing embarrassment.

14. (joy) 集団（個人）として、楽しみを経験しうる
    As a group, it is capable of experiencing joy.
    As an individual, one is capable of experiencing joy.

15. (fear) 集団（個人）として、恐怖を経験しうる
    As a group, it is capable of experiencing fear.
    As an individual, one is capable of experiencing fear.

16. (hesitation) 集団（個人）として、迷いやためらいを経験しうる
    As a group, it is capable of experiencing hesitation.
    As an individual, one is capable of experiencing hesitation.

17. (upset) 集団（個人）として、動揺を経験しうる
    As a group, it is capable of experiencing upset.
    As an individual, one is capable of experiencing upset.

doi: 10.3389/fpsyg.2019.00840

18. (anger) 集団（個人）として、怒りを経験しうる
    As a group, it is capable of experiencing anger.
    As an individual, one is capable of experiencing anger.

19. (sad) 集団（個人）として、悲しみを経験しうる
    As a group, it is capable of experiencing sadness.
    As an individual, one is capable of experiencing sadness.

20. (pride) 集団（個人）として、誇りを持ちうる
    As a group, it is capable of having pride.
    As an individual, one is capable of having pride.

21. (desire) 集団（個人）として、なにかを望み、求めることができる
    As a group, it is capable of wanting and hoping for and things.
    As an individual, one is capable of wanting and hoping for things.

22. (personality) 集団（個人）として、他と区別されるような個性を持ちうる
    As a group, it is capable of having personality traits that make it unique from others.
    As an individual, one is capable of having personality traits that make one unique from others.

Supplement 3: Factor analyses for mind attribution ratings

We conducted exploratory factor analyses (EFAs) separately on the ratings of mind attribution items for club, club members, company, and company members. All analyses used the maximum likelihood method and promax rotation.

First, we conducted EFA on the ratings for the club. Eigenvalues were 9.88, 3.10, 1.31, 0.95, 0.88, … in descending order, indicating that a two-factor structure was suitable. Thus, we conducted EFA to extract two factors; the factor loadings appear in Table S1.

Second, EFA was conducted on the ratings for individual club members. Eigenvalues were 6.96, 3.12, 2.49, 1.25, 1.06, 0.87, … in descending order, indicating that a three-factor structure was suitable. We conducted EFA to extract three factors (Table S1).

A third EFA was conducted on the ratings for the company. Eigenvalues were 9.67, 3.23, 1.13, 0.99, 0.91, …, indicating a two-factor structure. Thus, we conducted EFA to extract two factors (Table S1).

Finally, we conducted EFA on the ratings for the individual company members. Eigenvalues were 6.80, 2.96, 2.03, 1.34, 1.17, 0.95, …, indicating a three-factor structure. We conducted EFA to extract three factors (Table S1).

These analyses suggested there were overall common factor structures for both vignettes. The ratings for group mind attribution had a two-factor structure: one factor included thought, planning, memory, and so on, which covered the *agency* factor proposed by Gray et al. (2007). The other included feelings and sensations, such as pain, which covered the *experience* factor proposed by Gray et al. (2007). Meanwhile, the ratings for individual group members had a three-factor structure. Two of those three factors overlapped with the factors of group mind. The other factor consisted of cognition of others' feelings and intentions as well as communicating, indicating the capacity to infer inner state of other entities and interact with them. We called this factor *other-recognition*.

Next, we composed indices that could be compared between the targets of attribution. To this end, we had to decide how to treat items that loaded on unintended or inconsistent factors.

First, there were items that loaded on different factors from those proposed in previous research. *Desire*, *pride* (in all four analyses), and *personality* (in three of the four analyses) loaded on the agency factor in the present analyses, although they were supposed to load on the experience factor based on Gray et al. (2007). There could have been problems in the wording of the questionnaire, and these items might not have

measured the intended concepts. For example, the item for *desire* was "*wanting and hoping for and things*"; participants might have interpreted this item as similar to intentionality or goals, which can be included in agency. Thus, we excluded these three items from the analysis to avoid ambiguous conceptualization.

Second, *hesitation* and *joy* loaded on different factors depending on the analysis. *Hesitation* loaded on the experience factor in three of the four analyses. *Joy* loaded on the experience and agency factors twice; for one of the latter (company members), however, it was difficult to say which factor best covered this item (factor loadings were .242 for agency and .241 for experience). Given these results, it should be said that *hesitation* and *joy* were basically included in the experience factor, although they might reflect the concept of agency to some degree. Therefore, we decided to use these items to compose the experience index in the subsequent analyses.

Given that the EFAs on the ratings for individual members' mind suggested an inclusion of an unpredicted factor of other-recognition, we additionally conducted confirmatory factor analyses (CFAs) to test whether the three-factor structure fits the data better than the two-factor structure of agency and experience. We conducted CFAs with two- and three-factor structures separately and compared their goodness of fit. In the models, we included nineteen items from the original scale (i.e., we excluded three items, *desire*, *pride*, and *personality* for the reasons described above) and assumed that each item loaded on only one of the two or three latent factors. The results are displayed in Table S2. The results showed that the three-factor structure fitted the data much better than the two-factor structure. Next, we conducted likewise CFAs on the ratings for group mind to examine whether the three-factor structure also fits the ratings for group mind. This examination is required to compose the mind attribution indices according to the same structure as the individual members' mind attribution and compare the attribution between the groups and members. The results revealed that the three-factor structure fitted the data better than the two-factor structure (Table S3). A caveat would be that the models in overall did not quite reach the level of fitness as conventionally recommended (e.g., CFI > .95 and RMSEA < .05). The objective here was to decide on a structure that allows for the testing of our focal hypotheses while retaining comparability with existing studies. So instead of building more complex models which could potentially contribute to the model's fit, we decided to adopt the three-factor structure that exhibited relatively better fit than the two-factor structure in both the group and member mind attributions. Thus, based on the results of both the EFAs and the CFAs, we composed indices in accordance with the three-factor structure of agency, other-recognition, and experience for the group

mind as well as individual member mind, and compared the mind attribution between the group and its members in the hypothesis testing.

Table S1 Factor loadings for mind attribution

	Club-group		Club-members			Company-group		Company-members		
	Agency	Experience	Agency	Other-recognition	Experience	Agency	Experience	Agency	Other-recognition	Experience
thought	**.906**	-.067	**.788**	-.098	-.058	**.858**	-.161	**.900**	-.172	-.152
decision-making	**.853**	.013	**.725**	-.237	-.106	**.846**	-.125	**.894**	-.100	-.175
planning	**.777**	-.061	**.632**	-.079	-.035	**.734**	.051	**.445**	.104	.178
reflect	**.768**	.019	**.601**	-.016	.063	**.821**	.035	**.371**	.190	.075
self-control	**.745**	-.054	**.710**	.164	-.118	**.698**	.111	**.306**	.293	.119
predict	**.582**	.021	**.439**	.013	.266	**.796**	.019	**.340**	.219	.015
memory	**.573**	.118	**.571**	-.079	.212	**.796**	-.109	**.455**	-.045	.080
morality	**.560**	-.172	**.653**	.043	-.039	**.323**	.047	**.580**	.006	.019
desire	**.740**	.104	**.663**	.101	.058	**.817**	-.071	**.461**	.124	.131
pride	**.551**	.328	**.443**	.070	.340	**.775**	.035	**.336**	.165	.135
personality	**.513**	.097	**.631**	.165	-.102	.289	**.295**	**.362**	.058	.137
intention-recognition	**.814**	-.089	.030	**.930**	.010	**.732**	.019	-.062	**1.019**	-.094
emotion-recognition	**.823**	-.106	.075	**.929**	-.037	**.688**	.100	-.006	**.929**	-.133
communicating	**.826**	-.016	-.110	**.479**	.061	**.893**	-.112	.047	**.547**	.038
joy	.300	**.494**	.067	.202	**.509**	**.681**	.175	**.242**	.217	.241
hesitation	.138	**.642**	-.031	.041	**.759**	**.443**	.351	.027	-.137	**.669**
anger	-.134	**.830**	-.113	.131	**.816**	-.024	**.786**	-.102	.004	**.951**
sad	.041	**.808**	-.155	.031	**.864**	.151	**.718**	-.039	-.033	**.863**
fear	-.163	**.789**	.054	-.175	**.726**	-.148	**.853**	.113	-.150	**.693**
upset	-.219	**.779**	-.014	-.022	**.820**	-.025	**.765**	.068	-.070	**.724**
embarrassment	.002	**.775**	.008	-.055	**.651**	-.116	**.753**	-.036	.062	**.847**
pain	.233	**.637**	.225	-.119	**.529**	.251	**.410**	.013	.115	**.644**

Table S2 Solutions of confirmatory factor analyses of the ratings for individual members' mind

	Club-members					Company-members				
	Agency	Experience	Agency	Other-recognition	Experience	Agency	Experience	Agency	Other-recognition	Experience
thought	.737 ***		.737 ***			.545 ***		.661 ***		
decision-making	.602 ***		.605 ***			.549 ***		.660 ***		
planning	.637 ***		.639 ***			.674 ***		.662 ***		
reflect	.700 ***		.699 ***			.587 ***		.572 ***		
self-control	.600 ***		.599 ***			.552 ***		.489 ***		
predict	.652 ***		.651 ***			.575 ***		.525 ***		
memory	.732 ***		.733 ***			.435 ***		.482 ***		
morality	.602 ***		.601 ***			.561 ***		.609 ***		
intention-recognition	.109			.903 ***		.492 ***			.995 ***	
emotion-recognition	.122			.976 ***		.473 ***			.893 ***	
communicating	-.080			.448 ***		.440 ***			.553 ***	
joy		.525 ***			.524 ***		.389 ***			.387 ***
hesitation		.761 ***			.761 ***		.652 ***			.654 ***
anger		.748 ***			.747 ***		.904 ***			.903 ***
sad		.771 ***			.770 ***		.835 ***			.834 ***
fear		.746 ***			.747 ***		.713 ***			.715 ***
upset		.833 ***			.833 ***		.742 ***			.744 ***
embarrassment		.674 ***			.675 ***		.845 ***			.844 ***
pain		.632 ***			.633 ***		.676 ***			.675 ***
Correlations between factors										
Agency	1		1			1		1		
Other-recognition	-	-	.112	1		-	-	.301 **	1	
Experience	.417 ***	1	.419 ***	.015	1	.424 ***	1	.423 ***	.119	1
AIC	5194.3		5001.8			5118.4		4930.0		
CFI	.650		.823			.654		.820		
RMSEA	.150		.108			.150		.109		

Note. Numbers in the table are completely standardized solutions. Statistical significances are marked as ***p < .001 and **p < .01.

Table S3 Solutions of confirmatory factor analyses of the ratings for group mind

	Club					Company				
	Agency	Experience	Agency	Other-recognition	Experience	Agency	Experience	Agency	Other-recognition	Experience
thought	.884 ***		.885 ***			.817 ***		.818 ***		
decision-making	.878 ***		.875 ***			.824 ***		.806 ***		
planning	.744 ***		.769 ***			.739 ***		.759 ***		
reflect	.770 ***		.781 ***			.823 ***		.839 ***		
self-control	.694 ***		.682 ***			.741 ***		.736 ***		
predict	.595 ***		.628 ***			.808 ***		.821 ***		
memory	.639 ***		.660 ***			.754 ***		.786 ***		
morality	.463 ***		.463 ***			.328 ***		.344 ***		
intention-recognition	.759 ***			.922 ***		.739 ***			.933 ***	
emotion-recognition	.763 ***			.941 ***		.725 ***			.912 ***	
communicating	.807 ***			.709 ***		.865 ***			.753 ***	
joy		.665 ***			.663 ***		.488 ***			.492 ***
hesitation		.713 ***			.713 ***		.566 ***			.566 ***
anger		.745 ***			.746 ***		.775 ***			.775 ***
sad		.844 ***			.843 ***		.799 ***			.800 ***
fear		.681 ***			.682 ***		.745 ***			.743 ***
upset		.634 ***			.635 ***		.744 ***			.742 ***
embarrassment		.780 ***			.780 ***		.667 ***			.667 ***
pain		.758 ***			.759 ***		.541 ***			.542 ***
Correlations between factors										
Agency	1		1			1		1		
Other-recognition	-	-	.783 ***	1		-	-	.764 ***	1	
Experience	.509 ***	1	.514 ***	.386 ***	1	.412 ***	1	.414 ***	.396 ***	1
AIC	5438.5		5387.3			5561.4		5522.0		
CFI	.801		.837			.770		.798		
RMSEA	.131		.119			.140		.132		

Note. Numbers in the table are completely standardized solutions. Statistical significances are marked as ***p < .001.

Table S4 ANOVA for mind attribution

Factor	df	*F*	*p*	η_p^2
Entitativity	1, 112	86.97	< .001	.437
Dimension of mind	2, 224	4.91	.008	.042
Target of attribution (group vs. members)	1, 112	141.24	< .001	.558
Vignette (club vs. company)	1, 112	0.01	.922	< .001
Entitativity × Dimension	2, 224	40.53	< .001	.266
Entitativity × Target	1, 112	115.64	< .001	.508
Entitativity × Vignette	1, 112	2.33	.129	.020
Dimension × Target	2, 224	8.87	< .001	.073
Dimension × Vignette	2, 224	3.50	.032	.030
Target × Vignette	1, 112	10.21	.002	.084
Entitativity × Dimension × Target	2, 224	30.31	< .001	.213
Entitativity × Dimension × Vignette	2, 224	1.55	.214	.014
Entitativity × Target × Vignette	1, 112	1.56	.214	.014
Dimension × Target × Vignette	2, 224	2.56	.079	.022
Entitativity × Dimension × Target × Vignette	2, 224	1.85	.159	.016
